# Multi-Scale Safety Helmet Detection Based on RSSE-YOLOv3

**DOI:** 10.3390/s22166061

**Published:** 2022-08-13

**Authors:** Hongru Song

**Affiliations:** 1 College of Information Engineering, Zhejiang University of Technology, Hangzhou 310023, China; hongrusong@tlu.edu.cn; 2 College of Electrical Engineering, Tongling University, Tongling 244061, China

**Keywords:** YOLOv3, RSSE module, object detection, multi-scale

## Abstract

Due to the shielding of dense and small targets, real-time detection of whether construction workers are wearing safety helmets suffers from low detection accuracy and missed detection. In this paper, a new detection algorithm based on YOLOv3 is proposed. Firstly, the parallel network RepVGG Skip Squeeze Excitation (RSSE) module is used to replace the Res8 module in the original YOLOv3 network. The RSSE module consists of 3 × 3 convolutional fusion channels and SSE branches fused. The introduction of the R-SSE module increases the network width, reduces the network depth, and improves the network detection speed and accuracy. Secondly, to avoid gradient disappearance and improve feature reuse, the residual module Res2 is used to replace the CBL×5 modules. Finally, the resolution of the input image is improved, and the four-scale feature prediction is used instead of the three-scale feature prediction to further improve the efficiency of detecting small targets. This paper also introduces the complete joint crossover (*CIOU*) to improve the loss function and positioning accuracy. The experimental results show that, compared with the original YOLOv3 algorithm, the improved algorithm improves the precision (*P*) by 3.9%, the recall (*R*) by 5.2%, and the average precision (*mAP*) by 4.7%, which significantly improves the performance of the detection.

## 1. Introduction

With the increasing acceleration of urbanization, which has led to the rapid development of infrastructure construction, safe production is an important guarantee to promote growth. Safety helmets play an important role in ensuring safe production and can effectively protect the head safety of on-site construction workers, reducing the operational risk of construction workers to a certain extent. In the safe production specifications of construction and heavy industry, it is stipulated that a worker is not allowed to enter the construction site without wearing a safety helmet. However, in practice, construction employees often fail to wear safety helmets. To monitor and correct unsafe behavior and ensure the safety of construction workers, it is necessary to carry out real-time detection on whether construction workers are wearing safety helmets. On the construction site, there is often a situation where the construction personnel are far away from the monitoring equipment, which makes the monitoring target exhibit the characteristics of small-scale targets. Due to the few small-scale target pixels, inconspicuous image features, dense targets, and other reasons, small target missed detection and target occlusion is prone to occur in the detection [1,2]. Traditional helmet wearing detection mainly adopts manual inspection and video monitoring systems, with various defects such as low efficiency, high cost, limited management scope, and missed detection [3]. In response to the shortcomings of traditional detection methods, deep learning-based target detection algorithms have numerous advantages, such as fast detection speed and high accuracy. They have been widely used in wearing safety helmet detection. 

Currently, there are two main types of target detection algorithms based on deep learning, the two-stage algorithm based on the region candidate network (RPN) [4] to extract candidate target information, and the one-stage algorithm based on regression. Two-stage detection algorithms mainly include regional convolutional neural networks (RCNN) [5], fast R-CNN [6], faster R-CNN [7], and mask R-CNN [8], etc. One-stage detection algorithms mainly include the single shot multibox detector (SSD) [9], the multi-scale deconvolution single shot multibox detector (MDSSD) [10], and the You Only Look Once (YOLO) algorithm [11,12,13,14,15].

This paper mainly studies the problem of low detection accuracy caused by dense target occlusion, and small-scale target missed detection in helmet wearing detection. A new model is established by improving the YOLOv3 algorithm, and the feasibility of the new model is verified on the helmet dataset.

The contributions of this paper are as follows: (1) we propose a parallel RSSEnetwork module to replace the Res8 module in Darknet53. It can increase the network width, reduce the network depth, and improve the network speed and the detection accuracy of small targets in the model; (2) We propose using residual module Res2 to replace CBL×5 modules in the YOLOv3 algorithm. This scheme can avoid gradient disappearance and enhance the reuse of features, improving the accuracy of detecting dense target occlusion; (3) We propose increasing the resolution of the input image to 608 × 608, and increasing the output feature map from three-scale detection to four-scale detection, which can improve the accuracy of the model for small target detection; (4) The ablation experiments on the helmet dataset show that the RSSE-YOLOv3 algorithm proposed in this paper has improved the detection performance. Compared with the original YOLOv3 algorithm, the *mAP* is increased by 4.7%, the *P* is increased by 3.9%, and the *R* is increased by 5.4%.

The content of this paper is arranged as follows: Section 2 summarizes related work of existing research on deep neural network design for the task of wearing hard hat object detection. Section 3 introduces the innovations of the improved algorithm. Section 4 introduces the dataset and evaluation criteria. Section 5 introduces various experimental schemes and describes the experimental results in detail. Section 6 concludes this paper and proposes the following research directions.

## 2. Related Work

In view of the problems in the process of detecting wearing helmets, many scholars have done a lot of research work on target detection algorithms based on deep learning. Li et al. [16] proposed a method to improve the faster RCNN model, which enhances the faster RCNN algorithm by combining online complex sample mining and a multi-part combination of target detection framework, using multi-scale training and adding an anchor point strategy. It improves the detection performance of the occluded part of the helmet. However, there is a lack of research on the detection of long-distance small targets. Fang et al. [17] proposed an improved YOLOv2 wearing safety helmet detection algorithm, which improved the accuracy of the YOLOv2 network and combined the network compression using the lightweight network structure in MobileNet. However, there is no research on the detection of dense target occlusion. He et al. [18] proposed an improved YOLOv3 multi-scale feature prediction algorithm. To further improve the accuracy of small-scale target detection, the feature pyramid structure was extended from the three-scale to four-scale algorithm. Xu et al. [3] proposed an improved YOLOv3 wearing safety helmet detection algorithm by adding feature maps, using *GIOU* Loss as the bounding box loss, and adding Focal Loss to the loss function to improve the accuracy of small target detection. There is a lack of research on the occlusion detection of dense targets in the literature. Cheng et al. [19] proposed to improve YOLOv3-tiny by building a depthwise separable convolution and guiding the light sandglass-residual (SR) module of the channel attention mechanism to replace the original model. The convolutional layer was used to replace the two-scale feature prediction with three-scale feature prediction, and the improved spatial pyramid pooling (SPP) module was added to the feature extraction network to improve the detection accuracy of the helmet. There is no research on long-range targets and occluded targets in the literature. Yan et al. [20] proposed an improved wearing safety helmet detection algorithm based on YOLOv3. The algorithm improves the speed and accuracy of wearing helmet detection by increasing the size of the input image, using depthwise separable convolution instead of the traditional convolution in Darknet53, and multi-scale feature fusion structure, etc. Han et al. proposed helmet wearing detection method based on YOLOv5 [21], which uses YOLOv5 as the baseline, predicts the bounding boxes of smaller objects by adding the fourth scale, and adopts an attention mechanism in the backbone network, and other methods to improve the speed and accuracy of helmets detection. There is no research on dense object occlusion detection in the literature. 

From the above research results, it can be seen that most researchers can improve the detection accuracy in the research of helmet wearing detection, but there is a lack of further research on the problem that the detection accuracy is reduced due to the occlusion of dense targets and the missed detection of small targets.

## 3. Methodology

### 3.1. RSSE Block Design

In 2019, Tan et al. [22] proposed that the convolutional network could be effectively scaled by increasing the depth, width, or resolution of the network, and used these methods to improve the accuracy of the network model. Among them, increasing the network depth increased the suggestive power of the network and contributed to learning increasingly abstract features. However, if the network depth is too deep, this leads to more sequential processing and higher latency, which slows down the rapid response of the network. At the same time, increasing the network width can facilitate multi-scale processing.

**Figure 1:**
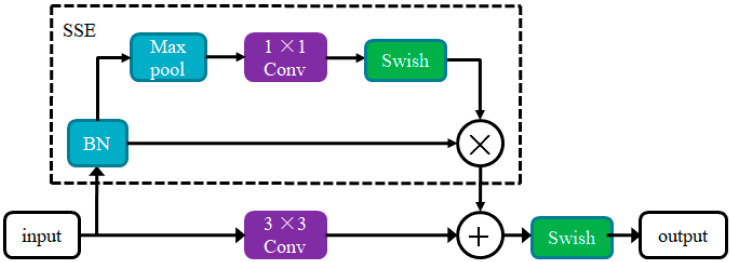
The RepVGG Skip Squeeze Excitation (RSSE) block.

In 2021, Goyal et al. [23] and others proposed the down-sampling RepVGG-SSE block, which is obtained by borrowing the initial block design of Rep-VGG [24] and modifying it to increase the network width to promote multi-scale processing, according to the structural principle of the Rep VGG-SSE block; this paper realizes the design of a parallel RSSE module. As shown in Figure 1, the RSSE module is divided into two parts. The lower part uses 3 × 3 convolution to fuse the channel information, which is added and fused with the output results of the upper Skip–Squeeze–Excitation (SSE) branch. The upper part is an SSE branch, which consists of a BN layer adding a single-layer SE module (Max pool, 1 × 1 Conv, Swish) in parallel with the connection branch. The main function of the SSE branch is to avoid a 3 × 3 convolution non-depth network, which can increase the receptive field without affecting the depth. Thus, the width of the network is increased, and the detection accuracy can be improved. In the design of the SSE branch, in order to retain more texture information in the detection target image during image feature extraction, the maximum pooling layer is used to realize the pooling function. At the same time, because the Swish function has the characteristics of no upper bound and lower bound, smoothness, and non-monotonicity, it can avoid gradient disappearance, and has better performance in the deep network model. The Swish activation function is used in the branch to improve the performance of the model.

As the Res8 module consists of a total of 25 layers of networks, the RSSE module consists of 8 layers of networks. In the improved algorithm, the RSSE module is used to replace the Res8 module in Darknet53 of the original YOLOv3 model, so that the backbone network of the YOLOv3 model is reduced from 74 layers to 52 layers, which reduces the depth of the network, and improves the running speed of the model.

### 3.2. Multi-Scale Detection Algorithm

In the case of the same input image, the smaller the size of the output feature map, the fewer grid cells of the image are segmented, the larger the image area contained in each grid cell, and the more feature extraction points, which will cause the omission of feature points. Conversely, the larger the size of the output feature map, the more grid cells the image is segmented, the smaller the image area contained in each grid cell, and the more accurate the feature extraction points. In the original YOLOv3 model, the input image size is 416 × 416, and the three output feature map sizes are 13 × 13, 26 × 26, and 52 × 52 for detecting large, medium, and small objects, respectively. This paper improves the detection accuracy of the YOLOv3 algorithm for small targets by increasing the model detection scale, so that the original three output detection layers are increased to four detection layers, and they are fused to realize the detection of small targets. The output feature map sizes of the detection layers are 13 × 13, 26 × 26, 52 × 52, and 104 × 104. At the same time, the CBL×5 unit modules in the original YOLOv3 model are replaced by Res2 modules to avoid gradient disappearance and enhance the reuse of features. It can improve the detection accuracy of small targets and occluded parts of dense targets.

**Figure 2:**
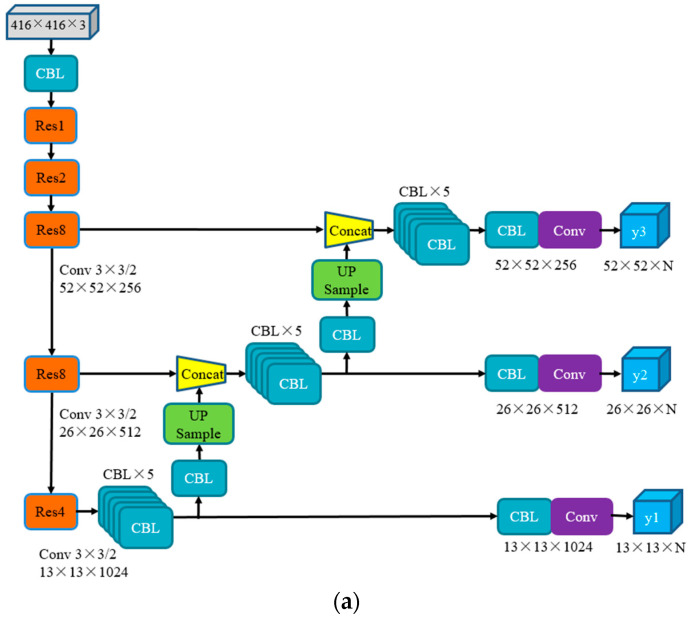

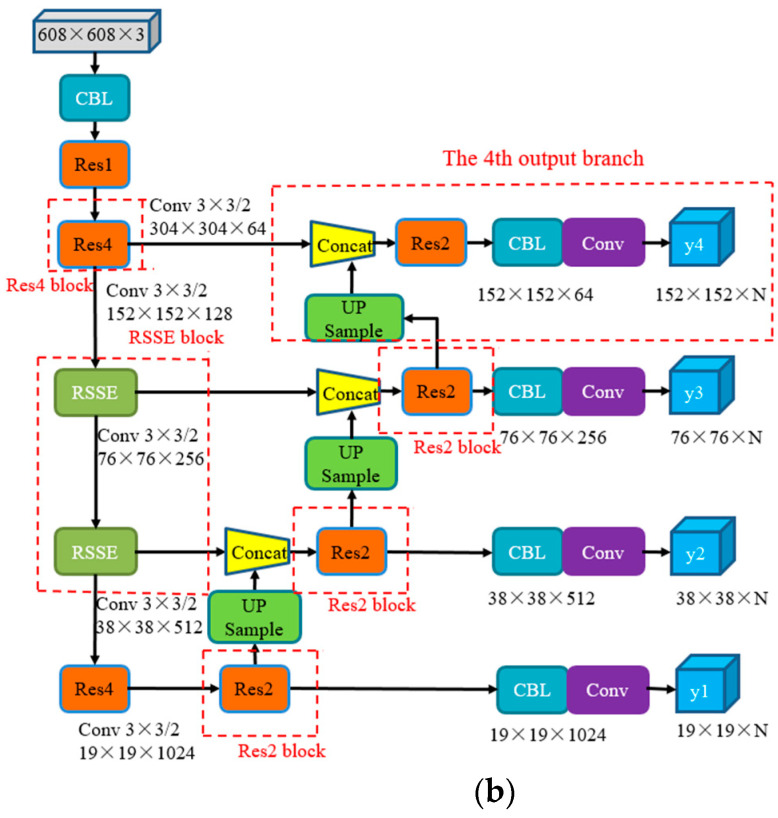
Comparison of network structure improvement. In order to highlight the improved part of the network structure, it is marked with a red box in (**b**). (**a**) The original YOLOv3 network structure; (**b**) The improved RSSE-YOLOv3 network structure.

Increasing the size of the input image can enhance the strength of extracting feature points and further improve the accuracy of detecting small objects. In this paper, the input image size is resized to 608 × 608, and the feature map sizes of the four detection layers are 19 × 19, 38 × 38, 76 × 76 and 152 × 152, respectively. The improved YOLOv3 model is referred to as RSSE-YOLOv3, as shown in Figure 2.

### 3.3. K-Means for Anchor Boxes

The YOLOv3 algorithm continues the method of YOLOv2 using the prairie box anchors to predict the coordinates of the bounding box, with the aim of having a larger *IOU* and a smaller distance between the anchor box and the adjacent ground truth box. Anchor values are calculated using the K-means clustering method [19,25].

The K-means algorithm selects K objects as the initial cluster center, calculates the distance between each object and the clustering center by using the metric formula D (Box, Centroid) = 1 − *IOU* (box, centroid), assigns the box to the clustering center with the closest “distance”(in the formula, d (box, centroid) denotes the distance from the anchor box to the cluster center; *IOU* (box, centroid) represents the intersection ratio of the anchor box and the ground truth box), and assign pairs according to the box. The cluster center points are recalculated for each cluster, and the clusters are re-clustered according to the dataset until all objects are classified. The experiment in this paper uses the self-built safety helmet dataset, so the original anchor box is no longer applicable and has to be re-clustered. Weighing the average intersection ratio and the number of cluster centers K, the number of clustering centers K is set to 9 for three detection layers, and the number of clustering centers K is set to 12 for four detection layers. When the input size is 608 × 608, under the prediction of three scales, take K = 9, the smallest 19 × 19 feature map has the largest receptive field, large anchors (143, 273), (229, 340), (379, 445) are used, which are suitable for detecting larger targets. On the medium 38 × 38 feature map, due to its medium receptive field, medium anchors (74, 120), (99, 194), (167, 162) are used, which are suitable for detecting medium-sized objects. With a larger 76 × 76 feature map, due to its smaller receptive field, the smallest anchors (11, 18), (25, 43), (44, 78) are used, which are suitable for detecting smaller targets. Similarly, under the prediction of four scales, take K = 12, the smallest 19 × 19 feature map has the largest receptive field, large anchors (129, 229), (188, 247), (291, 113) are used, which are suitable for detecting larger targets. On the medium 38 × 38 feature map, due to its medium receptive field, medium anchors (67, 130), (89, 180), (125, 129) are used, which are suitable for detecting medium-sized objects. On the larger 76 × 76 feature map, due to its smaller receptive field, smaller anchors (35, 58), (47, 89), (80, 82) are used, which are suitable for detecting smaller targets. On the larger 152 × 152 feature map, due to its smaller receptive field, the smallest anchors (6, 10), (14, 23), (22, 41) are used, which are suitable for detecting smaller targets. The candidate boxes after clustering are shown in Table 1.

### 3.4. Loss Function

In order to further improve the detection accuracy of the model, the complete joint crossover (*CIOU*) loss function is used to design the regression loss function of the target detection model. Compared with the calculation method of intersection over union (*IOU*) [26], *CIOU* [27] comprehensively considers the distance, overlap rate, scale and aspect ratio information between the target and the anchor frame, which can avoid the problem that the prediction frame does not intersect with the real frame, resulting in a loss function gradient of 0. Therefore, *CIOU* is more in line with the regression mechanism of the predicted box, making the generation of the bounding box more stable. The penalty term *CIOU* that minimizes the center point distance, overlap ratio, and aspect ratio is defined as follows:

(1) $$L_{CIOU}=1-IOU+\frac{\rho^{2}\left(b,b^{gt}\right)}{c^{2}}+\alpha v$$

(2) $$v=\frac{4}{\pi^{2}}{\left(\arctan\frac{w^{gt}}{h^{gt}}-\arctan\frac{w}{h}\right)}^{2}$$

(3) $$\alpha =\frac{v}{\left(1+IOU\right)+v}$$

In the above formula, *IOU* is the intersection over union; *b* and $b^{gt}$ represent the center point of the anchor box and the target box, respectively; *w*, *h* represent the width and height of the anchor box, respectively; $w^{gt}$, $h^{gt}$ represent the width and height of the target box, respectively; and $\rho \left(\cdot \right)=∥b-b^{gt}{∥}^{2}$ indicates the Euclidean distance. *c* is the diagonal length of the smallest enclosing box covering the anchor box and the target box. *α* is a balance factor, and *v* is a shape penalty term.

## 4. Experiments

In this paper, to train the defect detection proposed model, we used the framework of Darknet. The experimental computer configuration is an AMD Ryzen 7 5800X 8-core processor with CPU frequency of 3.80 GHz and RAM of 32 GB produced by AMD of the USA, and NVIDIA GeForce GTX 3060 graphics card with 12 GB of memory produced by NVIDIA of the USA.

### 4.1. Datasets

**Figure 3:**
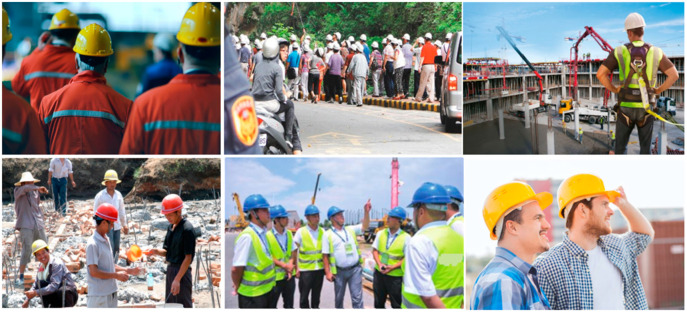
Dataset image.

At present, there is no unified dataset for wearing safety helmet detection. The test images used in this paper were from the open source VOC2012 dataset and SWHD, etc. Referring to the standards of the PASCAL VOC dataset, an image dataset containing multiple scenes and multiple targets was established, so that the detection model has detection ability in different scenes, so as to facilitate the model for training, testing, and analysis. This dataset was named the “safety helmet”. The dataset image is shown in Figure 3. The safety helmet data set was sorted and processed according to the research needs. The dataset used in this experiment contained 7500 pictures, which were saved in JPG format, labeled with labeling software, and saved as XML files. The labeled data were normalized and divided into a training set and test set according to the ratio of 8:2, and 6000 pictures were obtained as a training set and 1500 pictures as the test set.

### 4.2. Evaluation Criteria

In order to accurately and effectively evaluate the performance of the detection algorithm, this paper selected the accuracy rate *P*, recall rate *R*, and *mAP* as the evaluation indicators, and the calculation formula is:

(4) $$P=\frac{TP}{TP+FP}$$

(5) $$R=\frac{TP}{TP+FM}$$

(6) $$F1=\frac{2PR}{P+R}$$

(7) $$AP=\int_{0}^{1}P\left(R\right)dR$$

(8) $$mAP=\frac{\sum_{i=1}^{N}AP_{i}}{N}$$

In the above formula, *F*1 is the harmonic average of *P* and *R*, true positives (*TP*) is the number of correctly detected targets, false positives (*FP*) is the number of falsely detected targets, and false negatives (*FN*) is the number of undetected targets. Average precision (*AP*) indicates the detection effect of the algorithm on a target category. Mean average precision (*mAP*) represents the average accuracy of *N* categories.

## 5. Results and Discussions

### 5.1. Ablation Experiment

Due to the change in the original YOLOv3 network model structure, this paper used the method of random initial weights for model training. In the experiments of this paper, in order to ensure the effectiveness of the experimental comparison results and reflect the advanced nature of the improved algorithm model, all models were retrained in the same experimental environment to complete the model test. In the experiment, epochs was set to 200, the batch size was set to 32, the learning rate was changed from 0.01 to 0.00001, the momentum was set to 0.9, and the weight decay was set to 0.0005.

To better understand the detection effect of each improved method, ablation learning was performed on the safety helmet test set. Each improvement scheme is described in Table 2. By comparing the *P*, *R*, *F*1, *mAP* and *FPS* indicators produced by the experiments of different schemes, the performance of each scheme was comprehensively analyzed. Table 3 shows the results of ablation experiments with an input size of 416 × 416, and Table 4 shows the results of ablation experiments with an input size of 608 × 608.

Different improvement schemes are shown in Table 2. In Table 2, 3Scale represents three scale prediction methods, and 4Scale represents four scale prediction methods. Specifically, in the 3Scale + *CIOU* scheme, three scale prediction methods were used, and the *CIOU* loss function was used on the basis of the original YOLOv3 to improve the regression accuracy of the target and improve the detection accuracy of dense targets. In the 3Scale + *CIOU* + RSSE scheme, based on the 3Scale + *CIOU* scheme, the parallel network RSSE module was introduced to replace the Res8 module in Darknet53, which can increase the network width, reduce the network depth, and improve the network speed and target detection accuracy. In the 3Scale + *CIOU* + RSSE + Res2 scheme, based on the 3Scale + *CIOU* + RSSE scheme, the residual network Res2 was used to replace the CBL×5 module in the original YOLOv3, which can avoid gradient disappearance and enhance feature reuse, so as to improve the detection accuracy of dense target occlusion. In order to further improve the performance of small target detection, the original model was improved from three scale predictions to four scale predictions. The improved four scale prediction schemes were 4Scale + *CIOU* scheme, 4Scale + *CIOU* + RSSE scheme and 4Scale + *CIOU* + RSSE + Res2 scheme, respectively.

**Figure 4:**
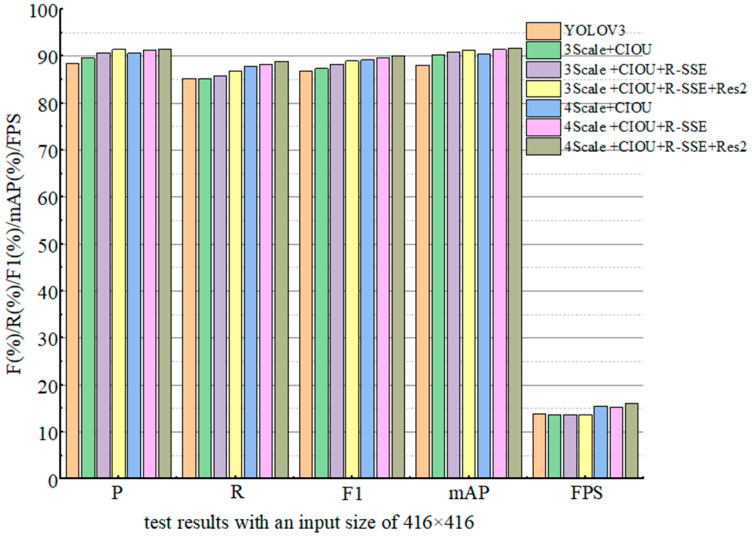
Histogram of test results with an input size of 416 × 416.

According to the different improvement schemes in Table 2, the ablation test results with an input size of 416 × 416 are shown in Table 3. In order to visually compare the results, a histogram was drawn according to the experimental data, as shown in Figure 4. It can be seen from Table 3 that in the original three-size YOLOv3 algorithm, the index values including *P*, *R*, *F*1, and *mAP* are relatively low. In the original YOLOv3 algorithm, *CIOU* loss function was used as the loss function, which increased the values of *P*, *F*1, and *mAP* by 1.2%, 1.5%, and 2.2%, respectively, while the test speed was basically unchanged. This shows that the introduction of the *CIOU* loss function can improve the regression accuracy of the model, and can effectively improve the detection accuracy of small targets. In the 3Scale + *CIOU* + RSSE scheme, the values of *P*, *F*1, and *mAP* were further improved by 1.1%, 0.9%, and 0.5%, respectively. This shows that the parallel network RSSE module can effectively improve the detection accuracy of small targets compared with the Res8 module. In the 3Scale + *CIOU* + RSSE + Res2 scheme, the values of *P, R, F1*, and *mAP* were increased by 0.7%, 0.9%, 0.7%, and 0.4%, respectively. This shows that the introduction of the residual module Res2 compared with the use of the CBL×5 module, the Res2 module can effectively improve the performance of the model to detect small objects. It can be seen from the above three improvement schemes that compared with the original YOLOv3 algorithm, the improved three-scale detection model algorithm increases the index values of *P*, *R*, *F*1, and *mAP* by 3%, 1.5%, 2.1% and 3.1%, respectively, while the *FPS* remains basically unchanged. This shows that the original YOLOv3 introduced the *CIOU* loss function, the parallel network RSSE module, and the staggered module Res2, which effectively improved the detection accuracy of small targets.

In the test using four scale algorithms, compared with the 3Scale + *CIOU* scheme, the *P*, *R*, *mAP*, and *F*1 of the 4Scale + *CIOU* scheme increased by 2.2%, 2.6%, 2.4%, and 2.4%, respectively, and the *FPS* was increased from 13.7 to 15.5. Compared with the 3Scale + *CIOU* + RSSE scheme, the *P*, *R*, *F*1, and *mAP* of the 4Scale + *CIOU* + RSSE scheme were increased by 0.6%, 2.4%, 1.5%, and 0.6%, respectively, and the *FPS* was increased from 13.6 to 15.3. Compared with the 3Scale + *CIOU* + RSSE + Res2 scheme, the 4Scale + *CIOU* + RSSE + Res2 scheme increased *P*, *R*, F1, and *mAP* by 0.1%, 2.1%, 1.2%, and 0.5%, respectively, and the *FPS* increased from 13.6 to 16.1. By comparing the experimental results of the three scale detection algorithms and the four scale algorithms in the several schemes, it can be seen that the four scale algorithms greatly improved the index values of *P*, *R*, *mAP*, *F*1, and *FPS*. The detection accuracy and detection speed of the model are improved. The experimental results show that the four detection scales have higher detection accuracy and faster detection speeds, and are more suitable for detecting smaller-sized targets.

**Figure 5:**
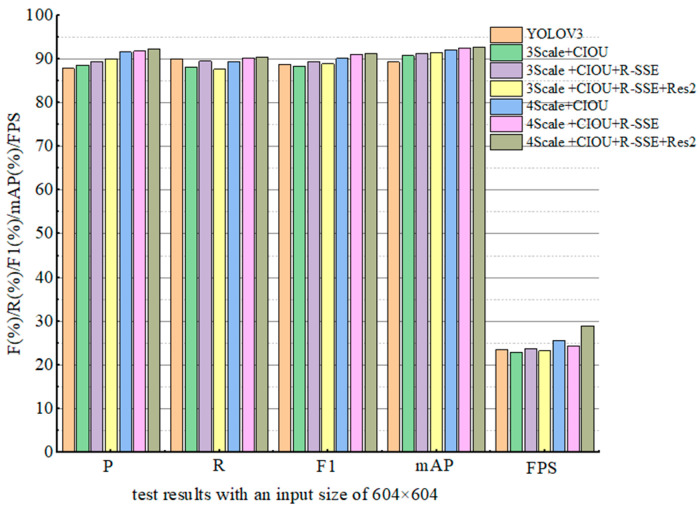
Histogram of test results with an input size of 604 × 604.

According to different improvement schemes in Table 2, the ablation experiment results with an input size of 608 × 608 are shown in Table 4. In order to visually compare the results, a histogram is drawn according to the experimental data, as shown in Figure 5. Comparing the experimental data in Table 3 and Table 4 under the same scheme, it can be seen that when the input size is 608 × 608, the values of *R*, *F*1, *mAP*, and *FPS* are improved when the *p* value slightly decreases in the test using the three scaling algorithms. In the test of four scale algorithms, all the index values have been improved; the value of *FPS* was improved most obviously, increasing from 16.1 to 28.9. This shows that the method of increasing the size of the input image can enhance the strength of the feature points extracted by the model, so as to further improve the accuracy of the detection target. From the comparison of the experimental results of the above improved schemes, it can be seen that the performance of the 4Scale + *CIOU* + RSSE + Res2 scheme with an input size of 608 × 608 has been greatly improved compared with the original YOLOv3. Specifically, it improves *P*, *R*, *mAP*, and *F*1 by 3.9%, 5.2%, 4.5%, and 4.7%, respectively, on the test set, and FPS from 13.8 to 28.9. The 4Scale + *CIOU* + RSSE + Res2 scheme with an input size of 608 × 608 was determined as the final improved algorithm (named RSSE-YOLOv3). The experimental results show that the final improved algorithm proposed in this paper not only improves the accuracy of detecting small targets, but also further improves the detection speed.

### 5.2. Result Comparison with Other Detection Models

**Figure 6:**
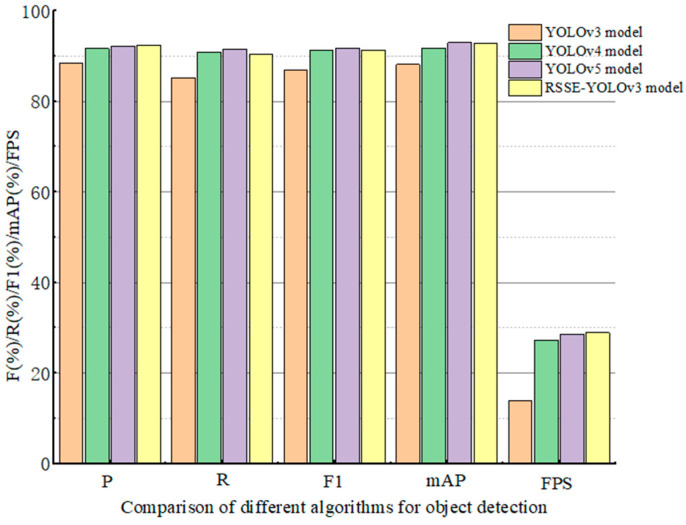
Comparison of histograms of test results of different algorithms.

The evaluation indicators of different detection models are analyzed and compared on the test set to prove the detection performance of each algorithm, as shown in Table 5. In order to visually compare the results, a histogram was drawn according to the experimental data, as shown in Figure 6. The following conclusions can be drawn from Table 5. Compared with YOLOv3 model, RSSE-YOLOv3 increased *P* from 88.4% to 92.3%, *R* from 85.2% to 90.4%, *F*1 from 86.8% to 91.3%, *mAP* from 88.1% to 92.8%, and *FPS* increased from 13.8 to 28.9. This shows that the improved RSSE-YOLOv3 model not only improves the speed of the network, but also improves the accuracy of detecting objects. Compared with YOLOv4 model, RSSE-YOLOv3 improved *P* from 91.6% to 92.3%, *FPS* from 27.3 to 28.9, and *mAP* from 91.8% to 92.8%, but *R* decreased. This shows that the improved RSSE-YOLOv3 model is better than the YOLOv4 model in detection accuracy and detection speed. Compared with the YOLOv5 model, the RSSE-YOLOv3 model improved *P* from 92.1% to 92.3%, and *FPS* from 28.5 to 28.9, but decreased *R* and *F*1, and *mAP* decreased by 0.3%. This shows that the RSSE-YOLOv3 model is lower than YOLOv5 model in detection accuracy, but has a slight advantage in detection speed.

**Figure 7:**
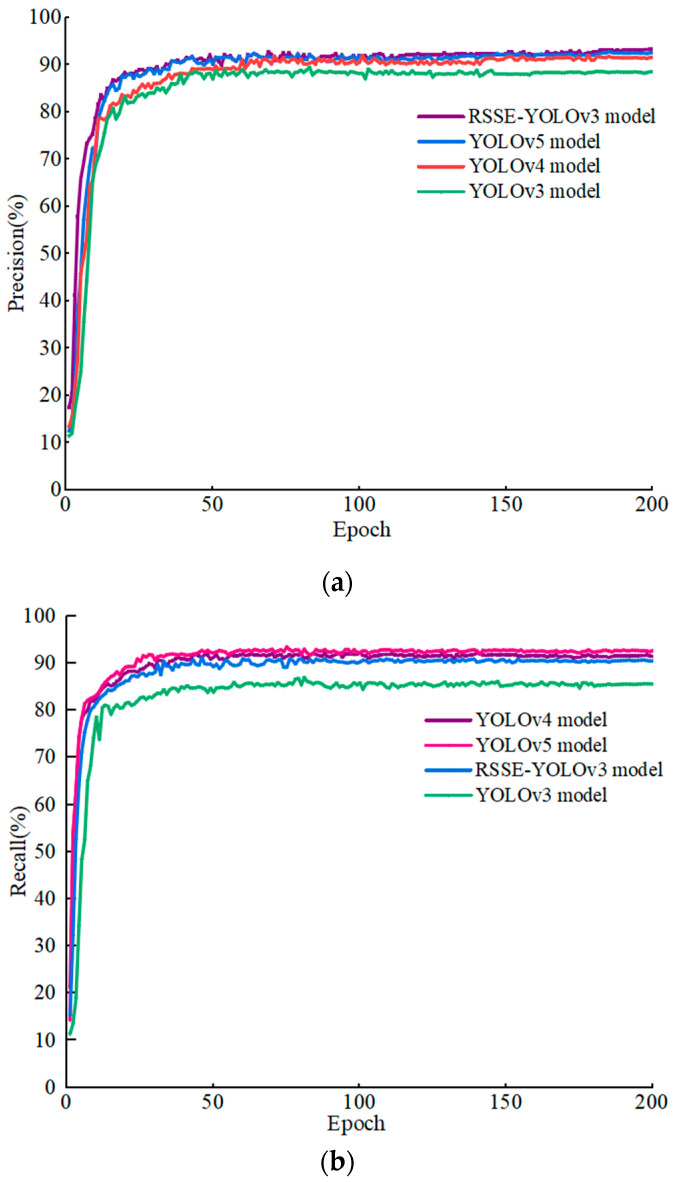
The curves of precision and recall in the different algorithms. (**a**) The curves of precision; (**b**) The curves of recall.

In order to further prove the effectiveness of different algorithms, according to the experimental values of the four algorithms, we draw two important performance indicators *P* and *R* curves. The curves of *P* and *R* are shown in Figure 7a,b. The horizontal axis in Figure 7a,b represents the training time, while the vertical axis represents the values of *P* and *R*, respectively. Comparing the *P* value curves in Figure 7a, it can be seen that the RSSE-YOLOv3 algorithm generates the highest *P* value, and the YOLOv3 algorithm generates the lowest *P* value. Comparing the R-value curves in Figure 7b, it can be seen that the R-value generated by the YOLOv5 algorithm is the highest, and the R-value generated by the YOLOv3 algorithm is the lowest. Although the R-value generated by the RSSE-YOLOv3 algorithm is lower than that of YOLOv4 and YOLOv5, it is higher than the R-value generated by YOLOv3. It can be obviously seen from Figure 7a,b that the RSSE-YOLOv3 algorithm is superior to the YOLOv3 algorithm.

### 5.3. Detection Results under Application Scenarios

**Figure 8:**
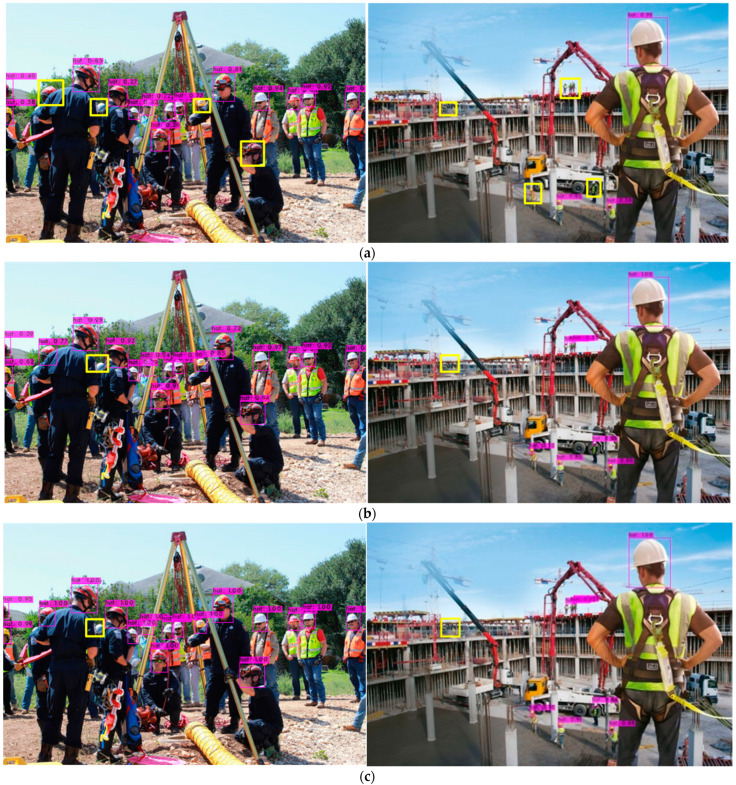

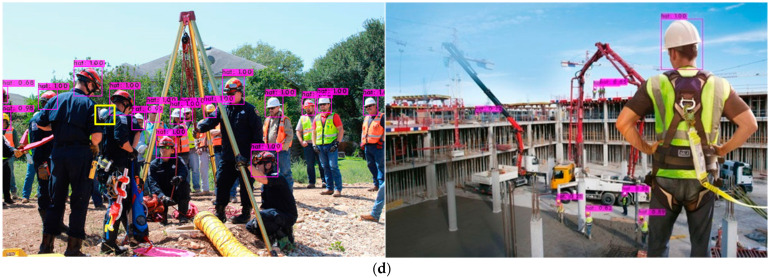
Comparison of detection results of different algorithms. (**a**) The detection result of the YOLOv3 model; (**b**) the detection result of the YOLOv4 model; (**c**) The detection result of the YOLOv5 model; (**d**) the detection result of theRSSE-YOLOv3 model. The object marked by the yellow box in the figure represents the missed detection target.

In order to further verify the detection effect of the algorithm in complex scenes, such as dense target occlusion and small targets, image detection was performed in the “safety helmet” dataset, and the detection effects of the YOLOv3 algorithm, RSSE-YOLOv3 algorithm, YOLOv4 algorithm, and YOLOv5 algorithm were compared. The comparison of the detection effects of the four algorithms is shown in Figure 8. In Figure 8, the left side shows the densely occluded target detection effect, and the right side shows the long-distance small target detection effect. It can be seen from the effect of the test images that the YOLOv3 algorithm has missed detection targets, and the accuracy is not high, especially for occluded targets and small targets. With the YOLOv3 algorithm, the detection target is less missed, and the accuracy is high. Compared with the YOLOv4 algorithm and the YOLOv5 algorithm, the RSSE-YOLOv3 algorithm has the same performance in the detection of dense targets and occluded targets. Although the accuracy rate is lower than the YOLOv4 algorithm and YOLOv5 algorithm, the detection effect of small targets is better. The detection results of four algorithms show that the RSSE-YOLOv3 algorithm significantly improves the detection performance of the model for dense targets, occluded targets, and small targets.

## 6. Conclusions

Considering the problem that the original YOLOv3 algorithm has low accuracy for dense target occlusion and small target detection, and is prone to miss detection, this paper proposes the RSSE-YOLOv3 algorithm. The algorithm adjusts the input size to 608 × 608, and adopts four scale prediction methods, which can enhance the strength of the feature points extracted by the model, thereby further improving the detection accuracy of small targets. During the ablation experiment, several experimental schemes were designed. In the 4Scale + *CIOU* scheme, based on the original YOLOv3, the *CIOU* loss function is used to improve the regression accuracy of the target, so as to improve the detection accuracy of dense targets. The 4Scale + *CIOU* + RSSE scheme is based on the 4Scale + *CIOU* scheme and uses the parallel network RSSE module to replace the Res8 module in Darknet53. The R-SSE module increases the network width of the model, reduces the network depth, and further improves the network detection speed and target detection accuracy of the model. The 4Scale + *CIOU* + RSSE + Res2 scheme is based on the 4Scale + *CIOU* + RSSE scheme, and the residual network Res2 is used to replace the CBL×5 modules in the original YOLOv3. The introduction of the Res2 module can avoid gradient disappearance and enhance feature reuse, and improve the detection accuracy of target occlusion and small target detection accuracy.

In conclusion, it can be seen from the experimental results of the test set that, compared with the original YOLOv3 algorithm, the RSSE-YOLOv3 algorithm has an increase of *P* from 88.4% to 92.3%, *R* from 85.2% to 90.4%, *F*1 from 86.8% to 91.3%, *mAP* from 88.1% to 92.8%, and *FPS* increased from 13.8 to 28.9. RSSE-YOLOv3 is inferior to YOLOv4 and YOLOv5 in detection accuracy, but it has advantages in speed. The comparison of experimental results shows that the RSSE-YOLOv3 algorithm improves the detection accuracy and speed of dense target occlusion and small target detection. Through the improvement of the YOLOv3 algorithm, this paper further improves the performance of the YOLOv3 algorithm for target detection, and provides theoretical support for the application of target detection technology in engineering. Future research work should further study the basic theoretical knowledge of YOLOv6/7 on the basis of YOLOv3 to improve the detection performance of YOLOv6/7 for small targets.

## Figures and Tables

**Table 1:** The candidate boxes after clustering.

K	Feature Map Size	Anchors		
K = 9	19 × 19	143, 273	229, 340	379, 445
	38 × 38	74, 120	99, 194	167, 162
	76 × 76	11, 18	25, 43	44, 78
K = 12	19 × 19	129, 229	188, 247	291, 113
	38 × 38	67, 130	89, 180	125, 129
	76 × 76	35, 58	47, 89	80, 82
	152 × 52	6, 10	14, 23	22, 41

**Table 2:** Different improvement schemes. “√” indicates the selected module.

Scheme	3Scale	4Scale	*CIOU*	RSSE	Res2
3Scale + *CIOU*	√		√		
3Scale + *CIOU* + RSSE	√		√	√	
3Scale + *CIOU* + RSSE + Res2	√		√	√	√
4Scale + *CIOU*	√		√		
4Scale + *CIOU* + RSSE		√	√	√	
4Scale + *CIOU* + RSSE + Res2		√	√	√	√

**Table 3:** Ablation results of different models (416 × 416).

Model	*P* (%)	*R* (%)	*F*1 (%)	*mAP* (%)	FPS
YOLOV3	88.4	85.2	86.8	88.1	13.8
3Scale + *CIOU*	89.6	85.2	87.3	90.3	13.7
3Scale + *CIOU* + RSSE	90.7	85.8	88.2	90.8	13.6
3Scale + *CIOU* + RSSE + Res2	91.4	86.7	88.9	91.2	13.6
4Scale + *CIOU*	90.6	87.8	89.2	90.5	15.5
4Scale + *CIOU* + RSSE	91.3	88.2	89.7	91.4	15.3
4Scale + *CIOU* + RSSE + Res2	91.5	88.8	90.1	91.7	16.1

**Table 4:** Ablation results of different models (608 × 608).

Model	*P* (%)	*R* (%)	*F*1 (%)	*mAP* (%)	FPS
YOLOV3	87.8	89.9	88.8	89.3	23.5
3Scale + *CIOU*	88.6	88.2	88.4	90.8	22.9
3Scale + *CIOU* + RSSE	89.3	89.5	89.4	91.2	23.7
3Scale + *CIOU* + RSSE + Res2	90	87.6	88.9	91.5	23.2
4Scale + *CIOU*	91.6	89.3	90.1	92.1	25.6
4Scale + *CIOU*+RSSE	91.8	90.2	91	92.5	24.3
4Scale + *CIOU* + RSSE + Res2	92.3	90.4	91.3	92.8	28.9

**Table 5:** Comparison of different algorithms for object detection.

Author	Model Used	*P* (%)	*R* (%)	*F*1 (%)	*mAP* (%)	FPS
Redmon et al. [13]	YOLOv3	88.4	85.2	86.8	88.1	13.8
Benyang et al. [28]	YOLOv4	91.6	90.8	91.2	91.8	27.3
Han et al. [21]	YOLOv5	92.1	91.5	91.8	92.9	28.5
Proposed model	RSSE-YOLOv3	92.3	90.4	91.3	92.8	28.9

## Data Availability

Some or all data, models or code generated or used during the study are available from the corresponding author by request.
